# Modeling perception and behavior in individuals at clinical high risk for psychosis: Support for the predictive processing framework

**DOI:** 10.1016/j.schres.2020.04.017

**Published:** 2020-12

**Authors:** Eren Kafadar, Vijay A. Mittal, Gregory P. Strauss, Hannah C. Chapman, Lauren M. Ellman, Sonia Bansal, James M. Gold, Ben Alderson-Day, Samuel Evans, Jamie Moffatt, Steven M. Silverstein, Elaine F. Walker, Scott W. Woods, Philip R. Corlett, Albert R. Powers

**Affiliations:** a Yale University School of Medicine and the Connecticut Mental Health Center, New Haven, CT, United States of America; b Northwestern University, Evanston, IL, United States of America; c University of Georgia, Athens, GA, United States of America; d Temple University, Philadelphia, PA, United States of America; e Maryland Psychiatric Research Center, Catonsville, MD, United States of America; f Durham University, Durham, UK; g University of Westminster, London, UK; h University of Sussex, Sussex, UK; i University of Rochester, Rochester, NY, United States of America; j Emory University, Atlanta, GA, United States of America

**Keywords:** Clinical high risk for psychosis, Computational psychiatry, Psychophysics, Predictive coding, Perception, Psychosis

## Abstract

Early intervention in psychotic spectrum disorders is critical for maximizing key clinical outcomes. While there is some evidence for the utility of intervention during the prodromal phase of the illness, efficacy of interventions is difficult to assess without appropriate risk stratification. This will require biomarkers that robustly help to identify risk level and are also relatively easy to obtain. Recent work highlights the utility of computer-based behavioral tasks for understanding the pathophysiology of psychotic symptoms. Computational modeling of performance on such tasks may be particularly useful because they explicitly and formally link performance and symptom expression. Several recent studies have successfully applied principles of Bayesian inference to understanding the computational underpinnings of hallucinations. Within this framework, hallucinations are seen as arising from an over-weighting of prior beliefs relative to sensory evidence. This view is supported by recently-published data from two tasks: the Conditioned Hallucinations (CH) task, which determines the degree to which participants use expectations in detecting a target tone; and a Sine-Vocoded Speech (SVS) task, in which participants can use prior exposure to speech samples to inform their understanding of degraded speech stimuli. We administered both of these tasks to two samples of participants at clinical high risk for psychosis (CHR; N = 19) and healthy controls (HC; N = 17). CHR participants reported both more conditioned hallucinations and more pre-training SVS detection. In addition, relationships were found between participants' performance on both tasks. On computational modeling of behavior on the CH task, CHR participants demonstrate significantly poorer recognition of task volatility as well as a trend toward higher weighting of priors. A relationship was found between this latter effect and performance on both tasks. Taken together, these results support the assertion that these two tasks may be driven by similar latent factors in perceptual inference, and highlight the potential utility of computationally-based tasks in identifying risk.

## 1 Introduction

Early detection and treatment of psychosis is critical for maintaining functionality and maximizing clinical outcomes (Kane et al., 2016; Srihari et al., 2012, Srihari et al., 2014). This effort has been made more reliable with the systematization of clinical evaluations for psychosis as it develops from the prodromal phase of the illness (Miller et al., 2002; Woods et al., 2009, Woods et al., 2014). Evaluation of progression continues to rely on symptom reports and clinical assessment. However, only a minority of those at clinical high risk of psychosis (CHR) will convert to frank psychosis (Hartmann et al., 2016) and the use of clinical measures alone, while promising, is nonetheless limited in predicting course and triggering treatment initiation (Cannon et al., 2016; Carrion et al., 2016). Development of objective measures for psychotic symptoms and disease states will be critical in identifying psychosis emergence, starting treatment early in the disease trajectory, and maximizing functionality in those affected.

Behavioral measures purporting to assess the cognitive and neural drivers of symptom expression may be sensitive and convenient measures of risk. Behavior on a number of tasks has thus far been linked to severity of specific psychotic symptoms, including hallucinations (Alderson-Day et al., 2017; Cassidy et al., 2018; Powers et al., 2017; Teufel et al., 2015), delusions (Corlett et al., 2007; Corlett and Fletcher, 2012), and positive, (Roiser et al., 2009, Roiser et al., 2013; Schmidt et al., 2017), disorganization (Silverstein et al., 2013; Silverstein and Keane, 2011; Uhlhaas et al., 2006), and negative symptoms (Gold et al., 2012; Heerey et al., 2007; Treadway et al., 2009) more broadly.

Measures derived from generative computational models linking behavior and symptom expression may hold particular promise as objective markers for psychiatric disease, in part because such models are capable of describing normal and pathological information processing within a common framework, capturing biology, behavior, and their pathology simultaneously (Browning et al., n.d.; Corlett and Fletcher, n.d.; Friston et al., 2014; Stephan and Mathys, 2014; Wang and Krystal, 2014). Here we utilize a predictive processing framework (Friston et al., 2006; K. Friston, 2005; Friston and Kiebel, 2009), which conceives of perception as the process of unconscious inference, in which we actively infer what is around us by combining our sensory input with our prior beliefs about the world (Friston, 2009). Within this framework, the brain functions as a predictive machine, predicting future states of the world using prior beliefs, which are then integrated with incoming sensory evidence to give rise to conscious perception.

Recent work has highlighted the utility of this predictive processing framework for understanding how specific alterations in learning and inference may produce the positive symptoms of psychosis (Adams et al., 2013b; Corlett et al., 2007, Corlett et al., 2010, Corlett et al., 2019; K. J. Friston, 2005; Powers et al., 2016; Sterzer et al., 2018). This has been especially true of hallucinations, which have been proposed to arise from inappropriate over-weighting of prior beliefs in perception (Corlett et al., 2019; Powers et al., 2016). Over several years, using multiple different methods, hallucinations have been related specifically to behavior signaling overly-precise priors (Alderson-Day et al., 2017; Cassidy et al., 2018; Powers et al., 2017; Zarkali et al., 2019). This appears to be true for hallucinations within the context of psychotic illness (Cassidy et al., 2018; Powers et al., 2017; Teufel et al., 2015) as well as in the general population (Alderson-Day et al., 2017; Powers et al., 2017), and for hallucinations arising from other neuropsychiatric disorders (Zarkali et al., 2019).

The utility of these behavioral measures as biomarkers may depend upon several as-yet-unknown factors. One such factor is theoretical: within the massive processing hierarchy of the brain, to what degree are these tasks and measures actually measuring the same latent construct? If the proposed abnormalities driving hallucinations (i.e., overly precise priors) are not unitary, the clinical utility of estimating them may be limited. Second, it is unclear whether hyper-precise priors are present not only in fully-formed hallucinations, but also in the earliest phases of illness. If not, the use of such measures to detect abnormalities leading to the expression of frank hallucinations may also be limited.

We present data derived from two tasks purporting to measure hyper-precise priors in hallucinations, collected in a sample of individuals across two CHR clinic sites, and among age-matched healthy controls. We demonstrate that both methods are sufficiently sensitive to detect hyper-precise priors in CHR and that their scores are correlated, supporting the hypothesis that these two methods reflect the same underlying construct. Lastly, we propose other computational parameters that may signal the need for care and increased risk for conversion in this high-risk group.

## 2 Methods

### 2.1 Participants

The sample comprised 19 CHR participants and 17 healthy controls (HC) recruited across two sites: the Georgia Psychiatric Risk Evaluation Program (G-PREP; directed by author G.P. Strauss) (CHR N = 9, HC N = 10) and the Adolescent Development and Preventive Treatment program (ADAPT; directed by author Vijay Mittal) (CHR N = 10, HC N = 7).

Similar recruitment procedures were followed across both sites, which involved referrals of youth displaying early signs of psychosis from local clinicians (e.g., psychiatrists, psychologists, social workers, school psychiatrists) to receive diagnostic assessment and monitoring evaluations. CHR youth were also recruited via online and print advertisements, and in-person presentations to community mental health centers.

### 2.2 Clinical procedures

The Structured Interview for Psychosis-Risk Syndromes (SIPS) (Miller et al., 1999) was administered to detect the presence of a psychosis-risk syndrome in three possible ways: 1) the presence of attenuated positive symptoms or fully psychotic positive symptoms occurring over a very brief time period; and/or 2) decline in global functioning accompanying the presence of schizotypal personality disorder and age < 19; and/or 3) a family history of schizophrenia with decline in functioning. The SIPS contains an instrument, the Scale of Prodromal Symptoms (SOPS), that rates the severity of relevant symptoms along a 7-point scale ranging from absent to severe and psychotic. Ratings in the range of 3 to 5 are required for designation as at CHR. This measure gauges several distinct categories of prodromal symptom domains including positive (unusual thoughts, suspiciousness, grandiosity, perceptual abnormalities, disorganized communication) and negative dimensions (social anhedonia, avolition, expression of emotion, experience of emotions and self, ideational richness, occupational functioning). The Structured Clinical Interview for the Diagnostic and Statistical Manual (SCID-I) (First et al., 1995) was administered to determine the presence of psychosis and substance dependence exclusionary criteria. Clinical interviews were conducted in person by advanced doctoral students, trained over a two-month period, and certified to perform the SIPS. All interviewers had inter-rater reliability scores that exceeded the minimum study criterion of Kappa >80.

Social functioning was assessed with the Global Functioning Scale: Social (GFS-S) (Carrión et al., 2019). This inventory provides ratings of functioning on a 10-point Likert scale where a score of 10 reflects “Superior Social/Interpersonal Functioning” and 1 indicates “Extreme Social Isolation”. The scale was designed for adolescents and has been found to be valid and reliable in assessing at-risk populations.

Healthy control (HC) participants were recruited from the local community using posted flyers and electronic advertisements. HC participants had no current major (former Axis I) DSM-5 diagnoses as established by the SCID (First, 2016). HC also had no family history of psychosis and were not taking psychotropic medications. All participants were free from lifetime neurological disease.

All participants provided written informed consent for a protocol approved by the University of Georgia and Northwestern University Institutional Review Boards and received monetary compensation for their participation.

### 2.3 Task procedures

Tasks were administered on Dell G3 15 gaming Laptops running Windows 10, MATLAB 2018b (www.mathworks.com), and the third iteration of the Psychophysics toolbox (http://psychtoolbox.org/). Responses were made by key press.

**Fig. 1:**
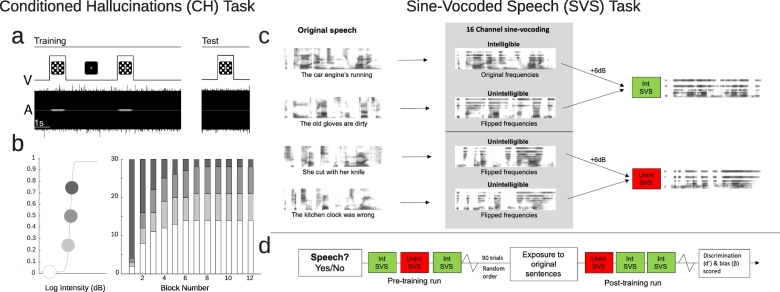
Behavioral tasks. The Conditioned Hallucinations (CH) task (a, b) and the Sine Wave Speech task (c, d) were administered. a. In the CH task, participants were asked to detect the presence of a 1-kHz tone embedded in white noise. The tone, when present, was paired with a white checkerboard flash on a black background, causing participants to build an association between the difficult-to-hear tone and salient checkerboard flash. b. We estimated individual psychometric curves for tone detection (left) and then systematically varied stimulus intensity over 12 blocks of 30 conditioning trials. Threshold tones were more likely early, and sub-threshold and absent tones were more likely later (right). c. Stimuli for the SVS task were created, of which some can become intelligible with training (top), and some are fully unintelligible (bottom). d. First participants are naive to the stimuli, and are asked to report their detection of speech for each trial. After training with the pre-degradation speech stimuli, participants repeat the task and are again asked to report their detection of speech.

Fig. 1 provides a schematic of the Conditioned Hallucinations (Fig. 1a, b) and the Sine Wave Speech (Fig. 1c) tasks.

### 2.4 Conditioned Hallucinations task

The Conditioned Hallucinations task (Powers et al., 2017) is an auditory detection task. Participants work to detect a tone (1 kHz) embedded in 70-dB SPL white noise and presented concurrently with a flashed gray checkerboard on a black background (Fig. 1a). Participants completed a short practice session reporting auditory detection, which was repeated until their responses were at 85% accuracy. Individual threshold (75% detection rate) is determined prior to the start of the experiment proper using the QUEST maximum likelihood-based procedure for threshold determination (Watson and Pelli, 1983), which is part of the Psychtoolbox 3.0 package in MATLAB. Thresholding was performed using two 40-trial interleaved staircases with step-sizes computed by QUEST during the participant responses. A psychometric function was fitted to the QUEST-computed 75% likelihood of detection of target stimulus embedded in noise (reported as dBSNR) (Treutwein and Strasburger, 1999), computing 50% and 25% detection-likelihood tone intensities (Fig. 1b, left). Total trial length during thresholding was 2500 ms.

In the experiment blocks, participants learned the association between the target auditory stimulus (tone) and a simultaneously presented visual stimulus (checkerboard). After this association-training, the participants were tested on this association over 12 blocks, with 30 trials each. The likelihood of tone presentation at threshold was decreased non-linearly over the 12 blocks, while increasing the presentation of subthreshold and no-tone trials (Fig. 1b, right). The trials were pseudorandomized within each block.

In addition to responding ‘Yes’ or ‘No’ to indicate whether or not they heard the tone, participants also reported their confidence level for their answer choice, by holding the response-button down; holding the button down longer indicated higher confidence in their decision of ‘Yes’ or ‘No’.

Throughout the experiment, a white visual fixation cross was present on a black background. The visual stimulus was a 4 × 7 gray-on-black checkerboard pattern, with gray squares at 25% brightness to maximize visual stimulation and minimize after-effect. The auditory stimulus was presented via Sony Professional MDR-7056 headphones, and consisted of a 1-kHz pure tone with a 100-ms tapered envelope to prevent transient effects.

For all parts of the experiment, there was a 500- to 1000-ms fixation from trial start, which was followed by the simultaneous presentation of the visual stimulus, and if present, the target auditory stimulus, for 1 s. Participant responses were recorded for 1000 to 1500 ms after stimulus offset. For the main part of the experiment, there was an additional 2000-ms period to record confidence-rating response, during which participants could hold down the response button to indicate their confidence level.

For both detection and confidence responses, if a response couldn't be reported, the trial was ignored and the stimulus intensity was repeated in the next trial. Fig. 1A shows specific stimulus characteristics described here, as well as the structure of a single trial. See Fig. 1A for a depiction of stimulus characteristics and trial structure.

### 2.5 Sine-Vocoded Speech task

Previous work using Sine Wave Speech (SWS) indicates that individuals that hallucinate are more likely to identify an ambiguous auditory stimulus as speech (Alderson-Day et al., 2017). Sine wave speech (SWS) is typically made by replacing the first three formants (main bands of energy) in speech with pure tones (Remez et al., 1981). It is often unintelligible on first exposure and may not even be recognized as speech-like (often sounding like ‘aliens’ or birdsong). Once the listener knows that it is potentially intelligible as speech (by training via exposure to pre-degradation speech templates, which thus serves as a prior expectation), relatively high levels of comprehension are achieved. Individuals who hallucinate are able to perceive speech in SWS even before exposure to the pre-degradation speech template and without being told speech is present (Alderson-Day et al., 2017), consistent with the presence of a strong prior for speech in people who hear voices.

Here, we used a similar signal manipulation, Sine-Vocoded Speech (SVS) (Souza and Rosen, 2009), that differs only in the respect that rather than tracking only the first three formants, sine waves are synthesised at the centre frequency of a bank of filters spanning a broad frequency range. With training, SVS sentences can be rendered intelligible and recognized as speech. SVS can also be rendered fully unintelligible by flipping the frequency mapping of the original sentence – providing an ideal control stimulus, with equal complexity (for full details on stimulus production, see Supplementary Materials) (Fig. 1c).

The “naïve” listening procedure can be challenging to reproduce due to the need to obscure the purpose of the task in advance. Here we therefore deployed a simpler paradigm assessing the ability of CHR participants to discriminate potentially intelligible SVS from unintelligible control SVS (45 trials/condition) before and after exposure to pre-degradation speech templates (i.e. updating their prior expectation). Participants were asked to report whether or not they detected speech on each trial (Fig. 1d).

In a pre-training phase, participants were presented with intelligible and unintelligible SVS and the number of correctly detected speech trials was recorded (hits) along with the number of unintelligible trials incorrectly classified as speech (false alarms). Following exposure to 90 trials (45 of each stimulus type), participants heard the 45 clear speech templates of the potentially intelligible SVS speech trials, before being tested on their SVS classification again.

### 2.6 Data analysis

#### 2.6.1 Conditioned Hallucinations task

We recorded: 1) participant responses for tone detection, 2) response times, 3) confidence rating levels. Trials with no recorded responses were discarded for the purposes of subsequent analyses. Detection probability was computed as the ratio of trials during which participants reported ‘Yes’ for hearing the tone, to those trials during which they reported ‘No’. No-tone trials where the participants recorded a ‘Yes’ response were considered conditioned hallucinations.

The Hierarchical Gaussian Filter (HGF) was fit to the behavioral data from the CH task. This model has been previously optimized specifically for use in the CH task, drawing upon evidence from simulations and Bayesian model comparison (Powers et al., 2017). The model is included in a freely-available toolbox (http://www.translationalneuromodeling.org/hgftoolbox-v4-10/). Details on the model are included in the Supplement.

#### 2.6.2 Sine Vocoded Speech task

We recorded the participants' responses for speech detection during both pre-training and post-training blocks. Using signal detection theory (Stanislaw and Todorov, 1999), we calculated participants' discrimination performance (d′), as well as their bias in classifying speech and non-speech (beta), and how those variables changed following the experience of template stimuli. One indication of enhanced speech priors is the detection of speech in unintelligible speech stimuli: a pre-training bias for speech. Another (following Teufel et al. (2015)) is any enhanced benefit of top-down information following template exposure, in the CHR relative to controls.

For both tasks, data from this small sample were not found to be normally distributed and nonparametric tests were used. Between-group differences for behavioral, as well as modeling variables were computed using Wilcoxon rank sum test with continuity correction. Correlations between measures were computed using Pearson's product-moment correlation and re-computed using Spearman's rank correlation to determine robustness to outliers. All analyses were done using packages *stats*, *tidyverse*, *tableone*, and plots were created using the *ggplot2* package, performed with the software RStudio 1.2.5001 (http://www.rstudio.com/).

## 3 Results

### 3.1 Sample characteristics

Table 1 summarizes the demographic features of the full healthy control (N = 17) and CHR (N = 19) samples. The groups were well-matched demographically, with the exception of a significant difference in racial makeup (χ^2^ = 13.814; p = 0.032). Clinical measures on the CHR and HC groups differed predictably. The CHR group had significantly higher P4 (SIPS Hallucinatory Behavior; T = 9.97, p < 0.001) and lower GAF scores (T = −7.90; p < 0.001) compared to matched healthy controls.

**Table 1:** Group demographic characteristics.

	CHR	HC	p
n	19	17	
Age (mean (SD))	20.95 (1.93)	20.88 (1.50)	0.911
Gender (portion male) (%)	5 (26.3)	1 (5.9)	0.232
Race (%)			0.065
African American	4 (21.1)	2 (11.8)	
Asian American	1 (5.3)	6 (35.3)	
Caucasian	12 (63.2)	5 (29.4)	
Latinx	2 (10.5)	1 (5.9)	
Multiracial	0 (0.0)	2 (11.8)	
Native American	0 (0.0)	1 (5.9)	
GAF Score (mean (SD))^a^	60.33 (11.08)	88.60 (5.46)	<0.001
WTAR/WRAT Score (mean (SD))^a^	106.78 (12.39)	112.88 (10.94)	0.14
LSHS Total Score (mean (SD))^b^	20.78 (8.29)	4.40 (6.02)	0.002
SIPS Positive Symptoms (mean (SD))^b^	12.11 (3.41)	0.29 (0.76)	<0.001
SIPS Negative Symptoms (mean (SD))^b^	5.33 (5.72)	1.14 (1.46)	0.071

a CHR N = 18; HC N = 5.

b CHR N = 18; HC N = 7.

Subsets consisting of individuals who performed the CH task (Table S1), the SVS task (Table S2), and both (Table S3) exhibited similar patterns of similarities and differences. CHR youth did not meet lifetime criteria for a DSM-5 psychotic disorder as determined via SCID interview (First et al., 1995). No CHR participants had been prescribed an antipsychotic.

### 3.2 Performance on both tasks differs between groups

**Fig. 2:**
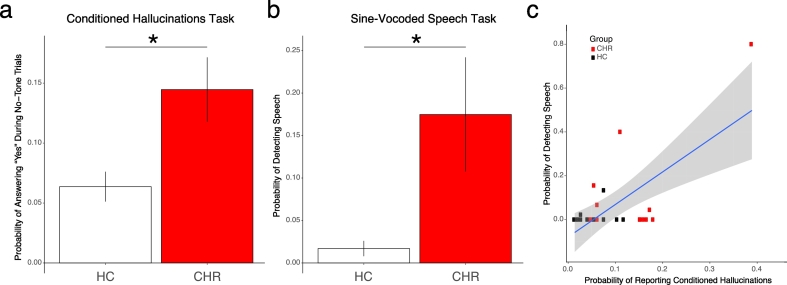
Group-level behavioral effects a. Mean between-group differences in CH task performance. CHR N = 12; HC N = 12. b. Mean between-group differences in SVS task performance. CHR N = 15; HC N = 19. c. Correlation between SVS task performance and CH task performance. CHR N = 11; HC N = 12. p < 0.001. Asterisk denotes p < 0.05.

As seen in Fig. 2a, CHR participants were more likely to report conditioned hallucinations (min = 0.04, max = 0.17; IQR = 0.11; median = 0.16; W = 119.5; p = 0.0066) than matched healthy controls (min = 0.01, max = 0.15; IQR = 0.056; median = 0.052).

Groups did not differ in initial threshold estimates (W = 49; p = 0.19; Fig. S1a). This was true only of the conditioned hallucination (no-tone) condition (Fig. S1b): group means did not differ at the 75% Detection (CHR median: 0.92; HC median: 0.94; W = 60.5; p = 0.52) or 50% Detection conditions (CHR median: 0.74; HC median: 0.81; W = 67; p = 0.79). The group difference in reporting detection at the 25% Detection condition trended toward significance (CHR median: 0.41; HC median: 0.28; W = 99.5; p = 0.12).

CHR participants were also more likely to exhibit pre-training detection of sine-wave speech (min = 0, max = 0.8; IQR = 0.28; median = 0.044; W = 175; p = 0.041; Fig. 2b) than healthy controls (min = 0, max = 0.13; IQR = 0; median = 0).

The behavioral effects for the SVS task reported above could be driven by differences between groups in latent variables, estimated using the Signal Detection Theory approach. During the pre-training portion of the task, the CHR group showed a higher mean bias for classifying the stimuli as speech (W = 73.5, p = 0.039). Measures of sensitivity did not change significantly after training (main effect of time: χ^2^ = 2.81; p = 0.070). There was also no significant difference between the groups post-training (W = 133; p = 0.84). There was a significant difference between CHR and healthy controls in change in pro-speech bias after training, with healthy controls exhibiting significantly more pro-speech bias after training (W = 38, p = 0.018) (Fig. S2).

We conducted an exploratory analysis to determine whether task performance correlated with symptom expression or performance on the other task. Both the pre-training speech bias (r = −0.513621, p = 0.017) and the change in speech bias after the training (r = −0.66, p = 0.0019) were significantly correlated with P4 (SIPS Hallucinatory Behavior) score. However, only the pre-post change in speech bias remained significant using an outlier-resistant correlation method (Spearman's rho = −0.75, p < 0.001).

Lastly, performance on both tasks correlated significantly (Fig. 2c; Pearson's R = 0.67; T_21_ = 4.1483; p = 4.56 × 10^−4^) but did not survive application of an outlier-resistant method (Spearman's rho: 0.19, p = 0.3758).

Interestingly, there was no correlation between P4 score and either the probability of reporting conditioned hallucinations (R = 0.178; p = 0.54) or pre-training detection of sine wave speech (R = 0.20; p = 0.39) within each group, or across the whole sample.

### 3.3 CHR participants differ in recognition of volatility in stimulus contingencies

**Fig. 3:**
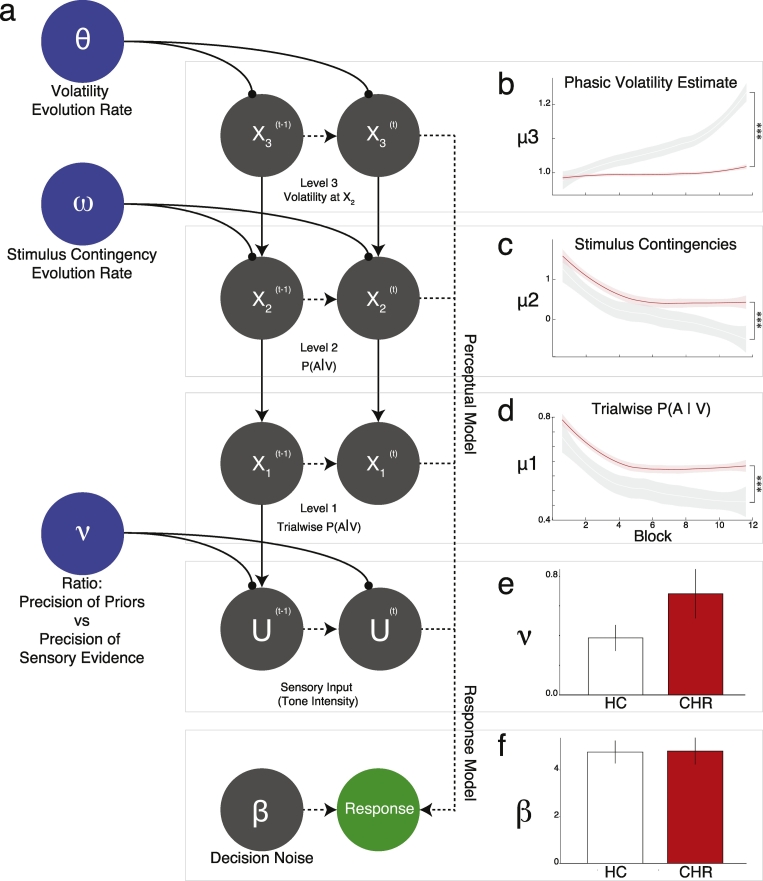
HGF model analysis. a. Schematic of the computation for the HGF model, mapping experimental stimuli to recorded responses. The first level (X1) represents whether the subject believes a tone was present or not on trial *t*. The second level (X2) is their belief that visual cues are associated with tones. The third level (X3) is their belief about the volatility of the second level. The HGF allows for individual variability in weighting between sensory evidence and perceptual beliefs (parameter *ν*). b. At X3, there was a significant block-by-group interaction. c–e. CHR participants also exhibited significantly higher beliefs in trial-wise (c) and experiment-long (d) contingencies between the presence of the tone and the visual stimulus. f. There were no inter-group effects of decision noise. ***P < 0.001; Error bars represent ±1 SEM. Line shadings represent 95% confidence intervals. Red = CHR; White with black outline = HC.

To provide further insight into the mechanisms driving the main behavioral effects above, we estimated parameters of a three-level Hierarchical Gaussian Filter (HGF; Fig. 3a) (Mathys et al., 2011; Stephan and Mathys, 2014) using behavior from the Conditioned Hallucinations task (Powers et al., 2017).

No difference in decision noise was seen between groups (Fig. 3f). CHR participants exhibited a trend toward higher relative precision of priors compared to healthy controls (Fig. 3e; W = 103; p = 0.078). Similar numerical differences were exhibited in terms of group belief trajectories: CHR participants tended to exhibit more tenacious beliefs that the tone was present when the visual stimulus was on any given trial (Fig. 3d) (χ^2^ = 12; p = 0.00053) and across the experiment (Fig. 3c) (χ^2^ = 12; p = 0.00053).

By contrast, groups did differ significantly in their ability to recognize the changing probabilistic relationship between the tone and the visual stimulus (Fig. 3b). While HC participants were likely to recognize that the visual stimulus became less predictive of the tone over time, CHR participants did not (χ^2^ = 8.33; p = 0.0039).

### 3.4 Prior precision correlates with performance on both tasks

**Fig. 4:**
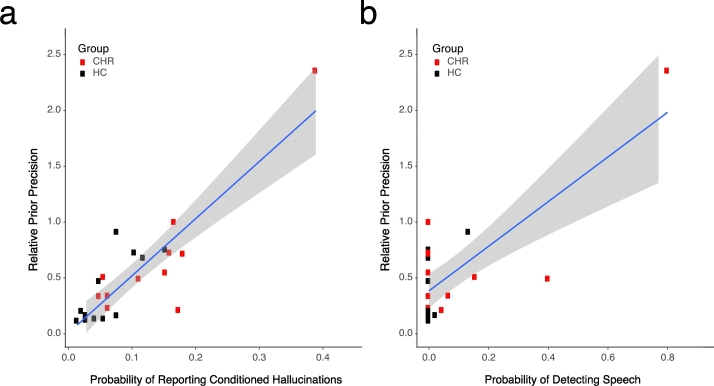
Relationships between prior precision and performance on both tasks. As expected, performance on the CH task (a) was predicted by prior precision. Performance on the separate Sine Wave Speech task (b) was driven by estimated prior precision on the CH task.

In order to determine whether prior precision drives performance, we explored whether a correlation existed between HGF-derived prior precision and performance on both tasks. As expected, estimated prior precision predicted performance on the Conditioned Hallucinations task (Fig. 4a; S = 507.74; rho = 0.75; p = 3.557 × 10^−5^). It also significantly predicted pre-training detection on the SVS task (Fig. 4b; R = 0.753; T_21_ = 5.245; p = 3.364 × 10^−5^), although this did not survive on non-parametric Spearman's correlation.

## 4 Discussion

We have demonstrated that participants at CHR for psychosis perform differently on two tasks grounded in predictive processing theory compared to healthy controls: CHR participants exhibited behavior consistent with hyper-precise priors on both tasks. Further, we have shown that performance on the two tasks are correlated, supporting some commonality of mechanism. Modeling of behavior on the Conditioned Hallucinations task using the HGF demonstrated group differences in volatility-related parameter estimates as well as a correlation between prior weighting and performance on both tasks.

The fact that CHR participants exhibited an increased tendency toward conditioned hallucinations as well as pre-training detection of sine wave speech indicates a tendency to exhibit hyper-precise priors even in the earliest phases of the illness. This is consistent with performance of at-risk individuals on recognition of previously-viewed visual scenes (Teufel et al., 2015). Interestingly, modeling of performance in the CHR group reflected that of individuals with psychosis and hallucinations in past work (Powers et al., 2017). Thus, model parameters demonstrated low change in X1, high relative prior precision, and low tendency to appropriately recognize volatility in the A-V contingency, although not all of these differences reached statistical significance in this small sample. This is consistent with the idea that the CHR condition may be accurately described as both an at-risk state and a syndrome conferring a need for care (Woods et al., 2001).

In the Conditioned Hallucinations task, participants are progressively exposed to fewer and fewer trials in which the target tone is predicted by the presence of the light. Thus, the contingency between the light and tone becomes progressively more volatile over the course of the experiment. Modeling of behavior using the HGF takes this volatility into account, explicitly estimating volatility beliefs related to the inter-stimulus contingency. It is particularly notable that inter-group differences in volatility estimates were even more significant than seen in individuals with fully-formed psychosis (Powers et al., 2017). The behavioral differences between groups in the SVS task shows that while the CHR group has a higher speech-bias before training, the HC group shows the ability to modulate speech-bias after acquiring new information in the post-training speech detection task, suggesting that the HC participants might have a more robust ability to modulate bias depending on environmental conditions compared to the CHR group. The results from both tasks are broadly consistent with recent accounts of volatility beliefs impacting low-level learning of action-outcome contingencies specifically in psychosis-spectrum illness (Deserno et al., 2020).

If computationally-oriented tasks are meant to assay the same underlying state in the same participants, and if this state is stable over time, performance on these tasks should be related. The results we present here support this relationship. For the first time, two tasks that have been thought to estimate the propensity of participants to rely upon their priors have been run on the same participants. Results show that this property appears to be conserved across tasks. Especially promising is the fact that estimated prior weighting on one task (CH) predicts performance on a separate task meant to assay the same underlying computational parameter, although these results should be interpreted with caution because of small sample size. Given that these are only two among several tasks to recently show prior-weighting effects in psychosis and psychosis-related states (Alderson-Day et al., 2017; Cassidy et al., 2018; Powers et al., 2017; Zarkali et al., 2019), it prompts the question as to whether all such measures capture the same latent state. Recent work has emphasized the need to take into account the hierarchical structure inherent in the systems involved (Corlett et al., 2019). Additionally, other models take explicit account of systems involved in action as they relate to perceptual inference (Adams et al., 2013a). Two such recent studies highlight the possibility that inference about action state (i.e., talking vs listening) may be a critical component of hallucinogenesis (Benrimoh et al., 2018, Benrimoh et al., 2019). It remains unclear whether and how tasks that purport to measure these computational alterations may themselves relate to the findings here. Future work should engage participants in a range of tasks meant to assay related model parameters, as well as subject data to several competing models, using principled means of comparison to determine the best explanatory fit for the data observed (Rosa et al., 2010).

The lack of correlation between symptom measures and performance on either tasks stands in stark contrast to the studies this work was based upon (Alderson-Day et al., 2017; Powers et al., 2017), which highlight a specific relationship to hallucinatory propensity in clinical and non-clinical voice-hearers. This lack of observed relationship may be due to several factors. First, there are statistical considerations: sample sizes from this study are approximately half those employed in the original studies, and only a small subset of individuals had self-reported hallucination severity measures; the range of symptom scores observed here is markedly low (P4 scores were clustered around 3 with low variation in the sample); and P4 values fail to take into account several phenomenological factors like frequency, intensity, or loudness of voice-hearing, instead focusing on more clinically-relevant factors such as distress or impairment associated with these experiences. Furthermore, the P4 measure does not take into account the sensory modality of the phenomenological experience, and whether the experience is visual or auditory could be important for mediating the performance on the auditory CH and SVS tasks. However, a second possibility may hold more promise for explaining pathological states leading to hallucinations. In this account, a lack of correlation with symptoms may be related to the phenomenologically semi-developed nature of the voice-hearing experience in CHR: most individuals with non-zero P4 scores experience relatively mild hallucinations, and most are non-verbal (Niles et al., 2019). This relative developmental nascency may mean that those who exhibit altered performance on these tasks and hyper-precise priors may not be individuals with high hallucination propensity at the moment, but may be more likely to develop frank hallucinations in the future. Longitudinal assessment of the relationship between prior precision and hallucinogenesis in CHR as well as symptomatic fluctuation in fully-formed psychosis is warranted to understand the clinical utility of the measures employed here. Further, larger multisite consortiums such as Computerized Assessment for Psychosis Risk (CAPR) and the proposed Psychosis Risk Outcome Network (PRONET) will be important for providing the statistical power and long-term clinical visit frequency density, necessary for model confirmation and refinement.

Taken as a whole, the findings presented here speak to the potential clinical utility and sensitivity of well-chosen behavioral measures, and–in particular–measures that are based solidly in explicit, formalized generative models for symptom expression. As a field, Computational Psychiatry comprises advocates for data-driven, machine-learning-based approaches to understanding heterogeneity of psychiatric presentation as well as others who espouse modeling as a way to uncover latent processes driving psychopathology (Browning et al., n.d.). The results here represent a way forward that marries the two approaches: employing measures that can be easily gathered from large, heterogeneous samples that nonetheless are able to speak to latent computational states that confer risk for symptom and disease progression. Future attempts may employ larger samples, with measures tied to other symptom domains, in an attempt to meaningfully parse the diversity of this markedly heterogeneous population. It may also be possible to employ both inferential and data-driven approaches within a hierarchical Bayesian framework, as has been done in model-based neuroimaging and electrophysiological approaches (Yao et al., 2018).

## Contributors

Tasks set up by ARP, PRC, BAD, SE, JM, and SB. Data were collected by team of VAM, GPS, HCC. Significant intellectual contributions from LME, JMG, SMS, EFW, SWW, PRC. Data analyzed, figures created, and manuscript written and edited by EK and ARP. Further edits from PRC, VAM, GPS, LME, JMG, AD, SE, and SMS.

## Role of the funding source

The research presented in this manuscript was supported by the funding sources named in the Acknowledgments section. These funding sources played no role in the collection, analysis and interpretation of data, in the writing of the report, or in the decision to submit the article for publication.

## Declaration of competing interest

We wish to confirm that there are no known conflicts of interest associated with this publication and there has been no significant financial support for this work that could have influenced its outcome.

## Appendix A Supplementary data

Supplementary material Image 1

## References

[bb0005] Adams R.A., Shipp S., Friston K.J. (2013). Predictions not commands: active inference in the motor system. Brain Struct. Funct..

[bb0010] Adams R.A., Stephan K.E., Brown H.R., Frith C.D., Friston K.J. (2013). The computational anatomy of psychosis. Front. Psychiatry.

[bb0015] Alderson-Day B., Lima C.F., Evans S., Krishnan S., Shanmugalingam P., Fernyhough C., Scott S.K. (2017). Distinct processing of ambiguous speech in people with non-clinical auditory verbal hallucinations. Brain.

[bb0020] Benrimoh D., Parr T., Vincent P., Adams R.A., Friston K. (2018). Active inference and auditory hallucinations. Comput Psychiatr.

[bb0025] Benrimoh D., Parr T., Adams R.A., Friston K. (2019). Hallucinations both in and out of context: an active inference account. PLoS One.

[bb0030] Browning, M., Carter, C., Chatham, C., den Ouden, H., Gillan, C., Baker, J., Goldstein, R., Lawson, R., Rindskopf, D., Roiser, J., n.d. Realizing the Clinical Potential of Computational Psychiatry: Report From the Banbury Center Meeting, February 2019. 10.1016/j.biopsych.2019.12.026 32113656

[bb0035] Cannon T.D., Yu C., Addington J., Bearden C.E., Cadenhead K.S., Cornblatt B.A., Heinssen R., Jeffries C.D., Mathalon D.H., McGlashan T.H., Perkins D.O., Seidman L.J., Tsuang M.T., Walker E.F., Woods S.W., Kattan M.W. (2016). An individualized risk calculator for research in prodromal psychosis. Am. J. Psychiatry.

[bb0040] Carrion R.E., Cornblatt B.A., Burton C.Z., Tso I.F., Auther A.M., Adelsheim S., Calkins R., Carter C.S., Niendam T., Sale T.G., Taylor S.F., McFarlane W.R. (2016). Personalized prediction of psychosis: external validation of the NAPLS-2 psychosis risk calculator with the EDIPPP project. Am. J. Psychiatry.

[bb0045] Carrión R.E., Auther A.M., McLaughlin D., Olsen R., Addington J., Bearden C.E., Cadenhead K.S., Cannon T.D., Mathalon D.H., McGlashan T.H., Perkins D.O., Seidman L.J., Tsuang M.T., Walker E.F., Woods S.W., Cornblatt B.A. (2019). The global functioning: social and role scales-further validation in a large sample of adolescents and young adults at clinical high risk for psychosis. Schizophr. Bull..

[bb0050] Cassidy C.M., Balsam P.D., Weinstein J.J., Rosengard R.J., Slifstein M., Daw N.D., Abi-Dargham A., Horga G. (2018). A perceptual inference mechanism for hallucinations linked to striatal dopamine. Curr. Biol..

[bb0055] Corlett P.R., Fletcher P.C. (2012). The neurobiology of schizotypy: fronto-striatal prediction error signal correlates with delusion-like beliefs in healthy people. Neuropsychologia.

[bb0060] Corlett, P.R., Fletcher, P.C., n.d. Computational psychiatry: a Rosetta Stone linking the brain to mental illness. Lancet Psychiatry 1, 399–402. doi:10.1016/S2215-0366(14)70298-6. 26361002

[bb0065] Corlett P.R., Murray G.K., Honey G.D., Aitken M.R., Shanks D.R., Robbins T.W., Bullmore E.T., Dickinson A., Fletcher P.C. (2007). Disrupted prediction-error signal in psychosis: evidence for an associative account of delusions. Brain.

[bb0070] Corlett P.R., Taylor J.R., Wang X.J., Fletcher P.C., Krystal J.H. (2010). Toward a neurobiology of delusions. Prog. Neurobiol..

[bb0075] Corlett P.R., Horga G., Fletcher P.C., Alderson-Day B., Schmack K., Powers A.R. (2019). Hallucinations and strong priors. Trends Cogn. Sci..

[bb0080] Deserno L., Boehme R., Mathys C., Katthagen T., Kaminski J., Stephan K.E., Heinz A., Schlagenhauf F. (2020). Volatility estimates increase choice switching and relate to prefrontal activity in schizophrenia. Biol Psychiatry Cogn Neurosci Neuroimaging.

[bb0085] First M.B. (2016). SCID-5-CV: Structured Clinical Interview for DSM-5 Disorders: Clinician Version.

[bb0090] First M.B., Spitzer R.L., Gibbon M., Williams J.B.W. (1995). The structured clinical interview for DSM-III-R personality disorders (SCID-II). Part I: description. J. Personal. Disord..

[bb0095] Friston K. (2005). A theory of cortical responses. Philos. Trans. R. Soc. Lond. Ser. B Biol. Sci..

[bb0100] Friston K.J. (2005). Hallucinations and perceptual inference. Behav. Brain Sci..

[bb0105] Friston K. (2009). The free-energy principle: a rough guide to the brain?. Trends Cogn. Sci..

[bb0110] Friston K., Kiebel S. (2009). Predictive coding under the free-energy principle. Philos. Trans. R. Soc. Lond. Ser. B Biol. Sci..

[bb0115] Friston K., Kilner J., Harrison L. (2006). A free energy principle for the brain. J. Physiol. Paris.

[bb0120] Friston K.J., Stephan K.E., Montague R., Dolan R.J. (2014). Computational psychiatry: the brain as a phantastic organ. Lancet Psychiatry.

[bb0125] Gold J.M., Waltz J.A., Matveeva T.M., Kasanova Z., Strauss G.P., Herbener E.S., Collins A.G.E., Frank M.J. (2012). Negative symptoms and the failure to represent the expected reward value of actions: behavioral and computational modeling evidence. Arch. Gen. Psychiatry.

[bb0130] Hartmann J.A., Yuen H.P., McGorry P.D., Yung A.R., Lin A., Wood S.J., Lavoie S., Nelson B. (2016). Declining transition rates to psychotic disorder in “ultra-high risk” clients: investigation of a dilution effect. Schizophr. Res..

[bb0135] Heerey E.A., Robinson B.M., McMahon R.P., Gold J.M. (2007). Delay discounting in schizophrenia. Cogn. Neuropsychiatry.

[bb0140] Kane J.M., Robinson D.G., Schooler N.R., Mueser K.T., Penn D.L., Rosenheck R.A., Addington J., Brunette M.F., Correll C.U., Estroff S.E., Marcy P., Robinson J., Meyer-Kalos P.S., Gottlieb J.D., Glynn S.M., Lynde D.W., Pipes R., Kurian B.T., Miller A.L., Azrin S.T., Goldstein A.B., Severe J.B., Lin H., Sint K.J., John M., Heinssen R.K. (2016). Comprehensive versus usual community care for first-episode psychosis: 2-year outcomes from the NIMH RAISE early treatment program. Am. J. Psychiatry.

[bb0145] Mathys C., Daunizeau J., Friston K.J., Stephan K.E. (2011). A bayesian foundation for individual learning under uncertainty. Front. Hum. Neurosci..

[bb0150] Miller T.J., McGlashan T.H., Woods S.W., Stein K., Driesen N., Corcoran C.M., Hoffman R., Davidson L. (1999). Symptom assessment in schizophrenic prodromal states. Psychiatr. Q..

[bb0155] Miller T.J., McGlashan T.H., Rosen J.L., Somjee L., Markovich P.J., Stein K., Woods S.W. (2002). Prospective diagnosis of the initial prodrome for schizophrenia based on the Structured Interview for Prodromal Syndromes: preliminary evidence of interrater reliability and predictive validity. Am. J. Psychiatry.

[bb0160] Niles H.F., Walsh B.C., Woods S.W., Powers A.R. (2019). Does hallucination perceptual modality impact psychosis risk?. Acta Psychiatr. Scand..

[bb0165] Powers, A.R., Iii, Kelley, M., Corlett, P.R., 2016. Hallucinations as top-down effects on perception. Biological Psychiatry: Cognitive Neuroscience and Neuroimaging 1, 393–400. doi:10.1016/j.bpsc.2016.04.003. PMC5469545 28626813

[bb0170] Powers A.R., Mathys C., Corlett P.R. (2017). Pavlovian conditioning-induced hallucinations result from overweighting of perceptual priors. Science.

[bb2005] Remez R.E., Rubin P.E., Pisoni D.B., Carrell T.D. (1981). Speech perception without traditional speech cues. Science.

[bb0175] Roiser J.P., Stephan K.E., den Ouden H.E.M., Barnes T.R.E., Friston K.J., Joyce E.M. (2009). Do patients with schizophrenia exhibit aberrant salience?. Psychol. Med..

[bb0180] Roiser J.P., Howes O.D., Chaddock C.A., Joyce E.M., McGuire P. (2013). Neural and behavioral correlates of aberrant salience in individuals at risk for psychosis. Schizophr. Bull..

[bb0185] Rosa M.J., Bestmann S., Harrison L., Penny W. (2010). Bayesian model selection maps for group studies. Neuroimage.

[bb0190] Schmidt A., Antoniades M., Allen P., Egerton A., Chaddock C.A., Borgwardt S., Fusar-Poli P., Roiser J.P., Howes O., McGuire P. (2017). Longitudinal alterations in motivational salience processing in ultra-high-risk subjects for psychosis. Psychol. Med..

[bb0195] Silverstein S.M., Keane B.P. (2011). Perceptual organization impairment in schizophrenia and associated brain mechanisms: review of research from 2005 to 2010. Schizophr. Bull..

[bb0200] Silverstein S.M., Keane B.P., Wang Y., Mikkilineni D., Paterno D., Papathomas T.V., Feigenson K. (2013). Effects of short-term inpatient treatment on sensitivity to a size contrast illusion in first-episode psychosis and multiple-episode schizophrenia. Front. Psychol..

[bb2010] Souza P., Rosen S. (2009). Effects of envelope bandwidth on the intelligibility of sine- and noise-vocoded speech. J. Acoust. Soc. Am..

[bb0205] Srihari V.H., Shah J., Keshavan M.S. (2012). Is early intervention for psychosis feasible and effective?. Psychiatr. Clin. North Am..

[bb0210] Srihari V.H., Tek C., Pollard J., Zimmet S., Keat J., Cahill J.D., Kucukgoncu S., Walsh B.C., Li F., Gueorguieva R., Levine N., Mesholam-Gately R.I., Friedman-Yakoobian M., Seidman L.J., Keshavan M.S., McGlashan T.H., Woods S.W. (2014). Reducing the duration of untreated psychosis and its impact in the U.S.: the STEP-ED study. BMC Psychiatry.

[bb2015] Stanislaw H., Todorov N. (1999). Calculation of signal detection theory measures. Behav. Res. Methods Instrum. Comput..

[bb0215] Stephan K.E., Mathys C. (2014). Computational approaches to psychiatry. Curr. Opin. Neurobiol..

[bb0220] Sterzer P., Adams R.A., Fletcher P., Frith C., Lawrie S.M., Muckli L., Petrovic P., Uhlhaas P., Voss M., Corlett P.R. (2018). The predictive coding account of psychosis. Biol. Psychiatry.

[bb0225] Teufel C., Subramaniam N., Dobler V., Perez J., Finnemann J., Mehta P.R., Goodyer I.M., Fletcher P.C. (2015). Shift toward prior knowledge confers a perceptual advantage in early psychosis and psychosis-prone healthy individuals. Proc. Natl. Acad. Sci. U. S. A..

[bb0230] Treadway M.T., Buckholtz J.W., Schwartzman A.N., Lambert W.E., Zald D.H. (2009). Worth the “EEfRT”? The effort expenditure for rewards task as an objective measure of motivation and anhedonia. PLoS One.

[bb0235] Treutwein B., Strasburger H. (1999). Fitting the psychometric function. Percept. Psychophys..

[bb0240] Uhlhaas P.J., Phillips W.A., Mitchell G., Silverstein S.M. (2006). Perceptual grouping in disorganized schizophrenia. Psychiatry Res..

[bb0245] Wang X.J., Krystal J.H. (2014). Computational psychiatry. Neuron.

[bb0250] Watson A.B., Pelli D.G. (1983). QUEST: a Bayesian adaptive psychometric method. Percept. Psychophys..

[bb0255] Woods S.W., Miller T.J., McGlashan T.H. (2001). The “prodromal” patient: both symptomatic and at-risk. CNS Spectr.

[bb0260] Woods S.W., Addington J., Cadenhead K.S., Cannon T.D., Cornblatt B.A., Heinssen R., Perkins D.O., Seidman L.J., Tsuang M.T., Walker E.F., McGlashan T.H. (2009). Validity of the prodromal risk syndrome for first psychosis: findings from the North American Prodrome Longitudinal Study. Schizophr. Bull..

[bb0265] Woods S.W., Walsh B.C., Addington J., Cadenhead K.S., Cannon T.D., Cornblatt B.A., Heinssen R., Perkins D.O., Seidman L.J., Tarbox S.I., Tsuang M.T., Walker E.F., McGlashan T.H. (2014). Current status specifiers for patients at clinical high risk for psychosis. Schizophr. Res..

[bb0270] Yao Y., Raman S.S., Schiek M., Leff A., Frässle S., Stephan K.E. (2018). Variational Bayesian inversion for hierarchical unsupervised generative embedding (HUGE). Neuroimage.

[bb0275] Zarkali A., Adams R.A., Psarras S., Leyland L.-A., Rees G., Weil R.S. (2019). Increased weighting on prior knowledge in Lewy body-associated visual hallucinations. Brain Commun.

