# Supplementary material for: Modeling perception and behavior in individuals at clinical high risk for psychosis: Support for the predictive processing framework
Source: Schizophr Res. 2020 Dec;226:167–75. doi: 10.1016/j.schres.2020.04.017 (PMC7774587; doi:10.1016/j.schres.2020.04.017)
Supplement: Supplementary file 1 — Supplementary material [file mmc1.pdf]

Supplemental Materials

Table S1. Demographics of participants who completed the CH task.

	**CHR**	**HC**	**p**
**n**	12	12	
**Age (mean (SD))**	21.08 (2.07)	21.00 (1.13)	0.903
**Gender (portion male) (%)**	1 (8.3)	1 (8.3)	1
**Race (%)**			0.068
**African American**	0 (0.0)	1 (8.3)	
**Asian American**	0 (0.0)	4 (33.3)	
**Caucasian**	11 (91.7)	4 (33.3)	
**Latinx**	1 (8.3)	1 (8.3)	
**Multiracial**	0 (0.0)	1 (8.3)	
**Native American**	0 (0.0)	1 (8.3)	
**Education (mean (SD))**	14.67 (1.92)	15.17 (2.17)	0.556
**N On Antipsychotic**	0	0	NA
**GAF_Score (mean (SD))**	58.83 (11.59)	90.00 (NA)	NA
**SIPS_p4 (mean (SD))**	3.27 (1.27)	0.00 (0.00)	**0.001**

Table S2. Demographics of participants completing the SVS Task.

	**CHR**	**HC**	**p**
**n**	15	17	
**Age (mean (SD))**	20.87 (2.13)	20.88 (1.50)	0.981
**Gender (portion male) (%)**	4 (26.7)	1 (5.9)	0.259
**Race (%)**			**0.045**

**African American**	3 (20.0)	2 (11.8)	
**Asian American**	0 (0.0)	6 (35.3)	
**Caucasian**	11 (73.3)	5 (29.4)	
**Latinx**	1 (6.7)	1 (5.9)	
**Multiracial**	0 (0.0)	2 (11.8)	
**Native American**	0 (0.0)	1 (5.9)	
**Education (mean (SD))**	14.40 (1.84)	14.94 (2.05)	0.44

Table S3. Demographics of participants who participated in both CH and SVS Tasks.

	**CHR**	**HC**	**p**
**n**	11	12	
**Age (mean (SD))**	21.09 (2.17)	21.00 (1.13)	0.899
**Gender (portion male) (%)**	1 (9.1)	1 (8.3)	1
**Race (%)**			0.089
**African American**	0 (0.0)	1 (8.3)	
**Asian American**	0 (0.0)	4 (33.3)	
**Caucasian**	10 (90.9)	4 (33.3)	
**Latinx**	1 (9.1)	1 (8.3)	
**Multiracial**	0 (0.0)	1 (8.3)	
**Native American**	0 (0.0)	1 (8.3)	
**Education (mean (SD))**	14.55 (1.97)	15.17 (2.17)	0.481

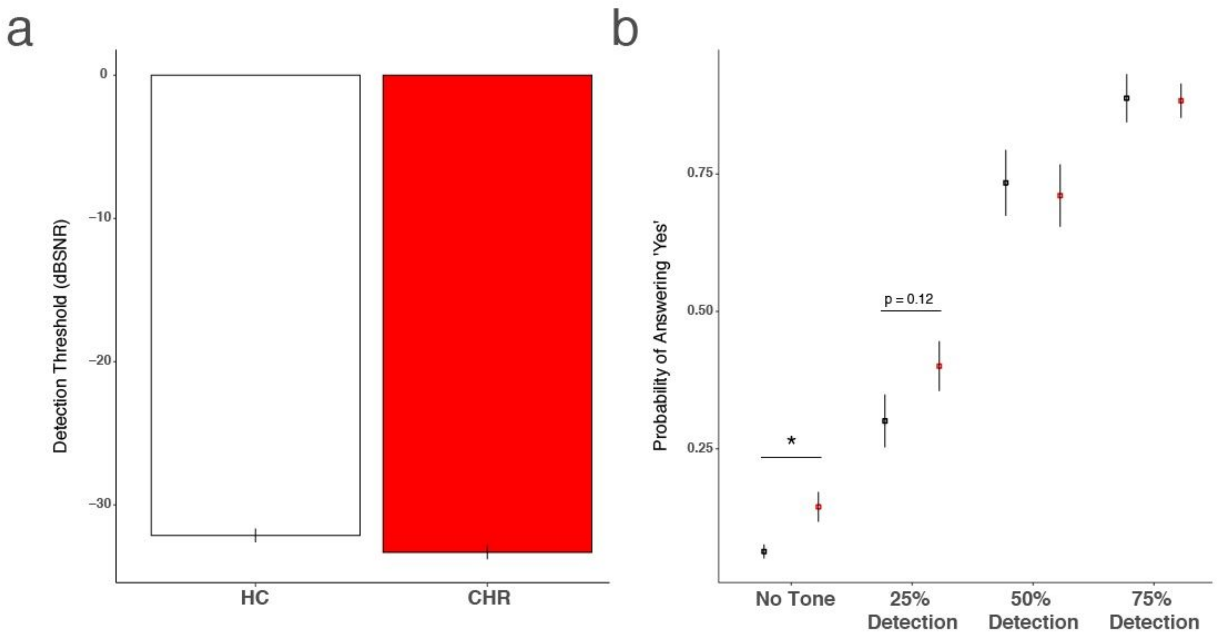

Figure S1. Supplemental CH results. a. CHR and HC participants did not demonstrate different thresholds for detection of the tone in white noise. **B.** Groups did not differ in their rates of tone detection at the 75% Detection, 50% Detection, or 25% Detection intensities, but only in the No-Tone condition.

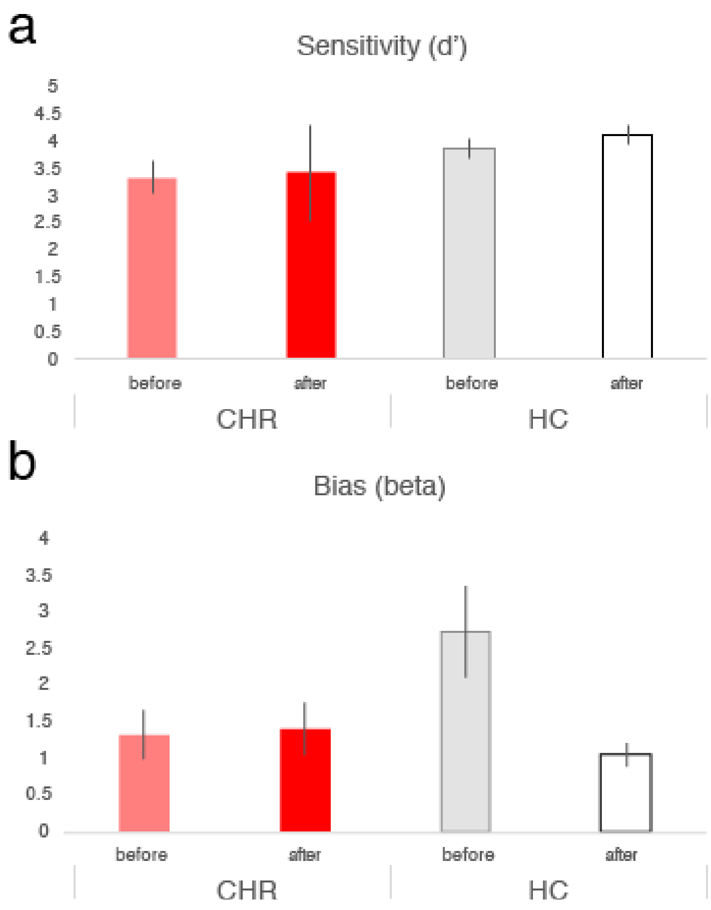

Figure S2. Supplemental SVS results. Signal-Detection Theory (SDT) analysis of sensitivity (**a**) and bias toward no voices being present (**b**).

Supplemental Methods.

Speech stimuli production

A male speaker was recorded at a sample rate of 22.05KHz producing the Bamford-Kowal-Bench sentences (Bench et al., 1979). Individual spoken sentences were digitally filtered using 16 bands, with sixth-order Butterworth IIR filters in MATLAB. Filter spacing was based on equal basilar membrane distance (Greenwood, 1990) across a frequency range of 100–5000 Hz. Next, the output of each band was half-wave rectified and low-pass filtered (fourth-order Butterworth) at 30 Hz to extract the amplitude envelope. The envelope was then multiplied by a tone carrier at the band center frequency for each filter. The resulting signal (envelope × carrier) was filtered using the same bandpass filter as for the first filtering stage. RMS level was adjusted at the output of the filter to match the original analysis, and the signal was summed across bands. This processing technique was used to make two types of sentences: intelligible and unintelligible SVS sentences.

For the Intelligible SVS condition, SVS sentences were further mixed with an unintelligible sine vocoded sentence to ensure sufficient difficulty of stimulus identification. The unintelligible sentence was sine-vocoded using the same signal processing as above, except that the bands were inverted such that the low frequency bands were shifted to high frequencies and vice versa, rendering it fully unintelligible. The relative RMS level of the intelligible sentence was fixed to be +6dB more intense than the unintelligible sentence before mixing them together. For the Unintelligible SVS condition, two sentences were sine-vocoded and frequency flipped and mixed together in the same ratio – in order to match the stimulus complexity of the intelligible SVS condition.

Hierarchical Gaussian Filter

The HGF is a general learning model that is based on discrete time intervals. It models inference on a dynamic environment based on consecutive input using hierarchical Bayesian computation. This model describes how an agent learns about continuous uncertainty that is dynamic over time, by mapping between implicit states of the world and the incoming sensory evidence. There are three states to the model (X1-X3), where the second and third levels evolve as hierarchical-coupled Gaussian random walks, while Bayes-optimal learning is approximated across levels.

Model parameters for the CH task were updated using tone intensities and participant responses from the individual trials. In this specific implementation of the model to the CH task data, decision noise (i.e., as a a logistic sigmoid, see below) was applied to to the probability of a “yes” response (to detection of tone) during a trial: $P(\text{"yes"}|belief) = sigmoid(belief)$ where we conceptualize *belief* as the posterior probability of a tone being present given the subject’s prior belief and the sensory intensity of the observed tone stimulus.

Belief is also formalized via the following Bayesian posterior mean of beta distribution

$$belief = prior + [1/(1 + v)]\,(observation - prior)$$

where *observation* depends on the experimental design: it is the expected detection rate for the tone in absence of the visual stimulus in individual trials: 25%, 50%, or 75%. The *prior* comes from the HGF model parameter $\hat{\mu}1$, and it is the prior belief due to learning the association between stimuli. ν is specific to each subject, and refers to the relative weighting of this prior compared to the *observation*. If $\nu = 1$, prior and observation are equally weighted; while when $\nu > 1$ the prior is weighted more than the observation; and for $\nu < 1$ the observation is weighted more than the prior.

The logistic sigmoid for the observation model is as follows:

$$f(x) = 1 \; e \; (-\beta*(V1 - V0))$$

where $V1$ and $V0$ respectively correspond to option 1 and 0 values, and $\beta > 0$ determines the shape of the sigmoid. Beta is sometimes considered as the (inverse) decision temperature, or inverse decision noise. Here, it represents the probability for choosing option 1. Probability of choosing option 0 is shown by interchanging $V1$ and $V0$. It has a necessary lower bound at zero, and is therefore estimated in log-space. $V1$ and $V0$ can be assigned any real number. Finally, the parameter Nu (ν), included in Figure 3A, was incorporated into second reworking of the model. The goal was to capture the tendency of individual participants' towards weighting priors more relative to sensory evidence, before making a decision.

As was done in (Powers et al. 2017), HGF generative models with fitted parameters were inverted to generate synthetic data. These were taken to be simulated responses of an observer with model parameters that had been estimated from behavior, and similarity of simulated responses to real responses are taken to be strong evidence of the ability of the model's parameters to account for the behavior observed. As was the case previously, there was an excellent correspondence between observed and simulated responses, producing 93.13% (+/- 0.08%) identical responses.
